# *In situ* small-angle X-ray scattering measurement at the Very Small Angle Neutron Scattering Instrument at the China Spallation Neutron Source

**DOI:** 10.1107/S1600576725001232

**Published:** 2025-02-28

**Authors:** Changli Ma, Xiong Lin, Zehuan Han, Songwen Xiao, Yongcheng He, Zhenqiang He, Fangwei Wang, He Cheng, Taisen Zuo

**Affiliations:** a Spallation Neutron Source Science Center, Dongguan 523803, People’s Republic of China; b Institute of High Energy Physics (IHEP) Chinese Academy of Science (CAS) Beijing 100049 People’s Republic of China; c School of Nuclear Science and Technology University of Chinese Academy of Sciences Beijing 100049 People’s Republic of China; d Institute of Physics Chinese Academy of Sciences Beijing 100190 People’s Republic of China; Argonne National Laboratory, USA

**Keywords:** very small angle X-ray scattering, SAXS, VSANS, vertical geometry, China Spallation Neutron Source

## Abstract

Small-angle X-ray and neutron scattering (SAXS and SANS) offer complementary insights into multi-scale and multiphase structures. Efforts have been made to integrate SAXS into SANS instruments, with only D22 at the Institut Laue–Langevin successfully implementing a SAXS setup; this was constrained to a horizontal geometry due to space limitations and high radiation noise. Here, we introduce an *in situ* vertical SAXS setup at beamline 14, Very Small Angle Neutron Scattering (VSANS) instrument, at the China Spallation Neutron Source. The compact vertical SAXS instrument without a beam stop, measuring 87 cm × 93 cm × 240 cm (W × L × H) and featuring a hoisting frame, can be easily installed in the VSANS sample room within 5 h. Utilizing a 50 mm-diameter neutron collimation guide, we can simultaneously detect X-rays from below and neutrons from behind, with the backboard of the SAXS chamber coated in a boron–aluminium alloy to reduce neutron background interference. Through testing with standard samples like deuterated and hydrogenated PEG, silver behenate, LaB_6_, and glass carbon, we demonstrate that concurrent neutron and X-ray measurements are successful.

## Abstract 2

A pioneering vertical-geometry small-angle X-ray scattering setup is available at beamline 14, Very Small Angle Neutron Scattering Instrument, at the China Spallation Neutron Source. Small-angle X-ray and neutron scattering can thus be performed at the same time.

## 1. Introduction

Small-angle X-ray and neutron scattering techniques (SAXS and SANS) are complementary approaches for studying hierarchical structures ranging from one to a few hundred nanometres (Zeng *et al.*, 2024 ▸; Hennig *et al.*, 2013 ▸; Koch *et al.*, 2003 ▸). In a SAXS experiment, X-rays interact with electrons outside the nucleus, so allowing the observation of structures of different electron density regions. The contrast in electron density between sample components can be altered by adjusting the solution density, such as by varying the salt concentration or adding sucrose or glycerol, to enable visualization of distinct structures (Grishaev *et al.*, 2012 ▸; Kuwamoto *et al.*, 2004 ▸; Jensen *et al.*, 2010 ▸; Putnam *et al.*, 2007 ▸; Arleth *et al.*, 2017 ▸; Blanchet & Svergun, 2013 ▸; Chen *et al.*, 2014 ▸; Gehrer *et al.*, 2014 ▸; Graceffa *et al.*, 2013 ▸; Liu *et al.*, 2011 ▸). In SANS experiments, neutrons interact directly with the nucleus to reveal structures on the basis of different neutron scattering lengths. Isotope substitution can modify the contrast between different structures in the sample. In particular, given that hydrogen and deuterium have significantly different scattering lengths (−3.739 and 6.671 fermi, respectively), altering the deuteration ratios of samples is a common method to adjust contrast in soft matter research. In summary, X-ray scattering mainly focuses on observing overall nanoparticle structures, while neutrons can reveal internal particle structure by utilizing different isotope selections (Zehua *et al.*, 2023 ▸; Han *et al.*, 2023 ▸; Lopez *et al.*, 2018 ▸; Weigandt *et al.*, 2011 ▸; Hennig *et al.*, 2013 ▸; Jordan *et al.*, 2016 ▸; Whitten *et al.*, 2008*a* ▸; Whitten *et al.*, 2008*b* ▸; Heller, 2010 ▸).

It is sometimes necessary to conduct joint measurements of X-ray and neutron scattering. For example, measuring neutron and X-ray scattering separately introduces a potential uncertainty for some samples, especially those like protein solution samples where ensuring complete consistency between duplicates is challenging. Conducting the two scattering experiments simultaneously is required in such cases. Another example is the exploration of non-equilibrium phenomena of polymer deformation. For decades, researchers were confused about the entangled chain orientation and stretching and their connection to shear overshooting. SAXS can ‘see’ the crystal structure, contrast-matching SANS can capture the chain structure, and their combination may reveal a microscopic picture of disentanglement. Therefore, setting up a SAXS environment in a SANS sample room holds significant value (Neylon, 2008 ▸; Schindler *et al.*, 2018 ▸; Schindler *et al.*, 2015 ▸; Schmiele *et al.*, 2014 ▸; Schmutzler *et al.*, 2018*a* ▸; Schmutzler *et al.*, 2018*b* ▸; Schmutzler *et al.*, 2019 ▸; Schuldes *et al.*, 2019 ▸; Spinozzi *et al.*, 2017 ▸; Svergun, 2010 ▸; Tanaka *et al.*, 2007 ▸).

Hence, significant efforts have been made to integrate the two detection methods. Yet, there are two primary challenges. The constrained space within the SANS sample room poses the primary obstacle. Following the removal of the sample stage, a typical sample room measures 2 m × 3 m, mirroring the dimensions of a standard horizontal-geometry pinhole SAXS instrument. Due to substantial shielding in a spallation source, there is limited space for manoeuvre. The second challenge pertains to radiation background. The X-ray detector is susceptible to neutron and gamma rays, with high-energy neutron and gamma rays capable of damaging its electronic components. To date, only the D22 facility at ILL has successfully implemented a horizontal-geometry SAXS measurement (Metwalli *et al.*, 2020 ▸; Schindler *et al.*, 2015 ▸).

**Figure 1:**
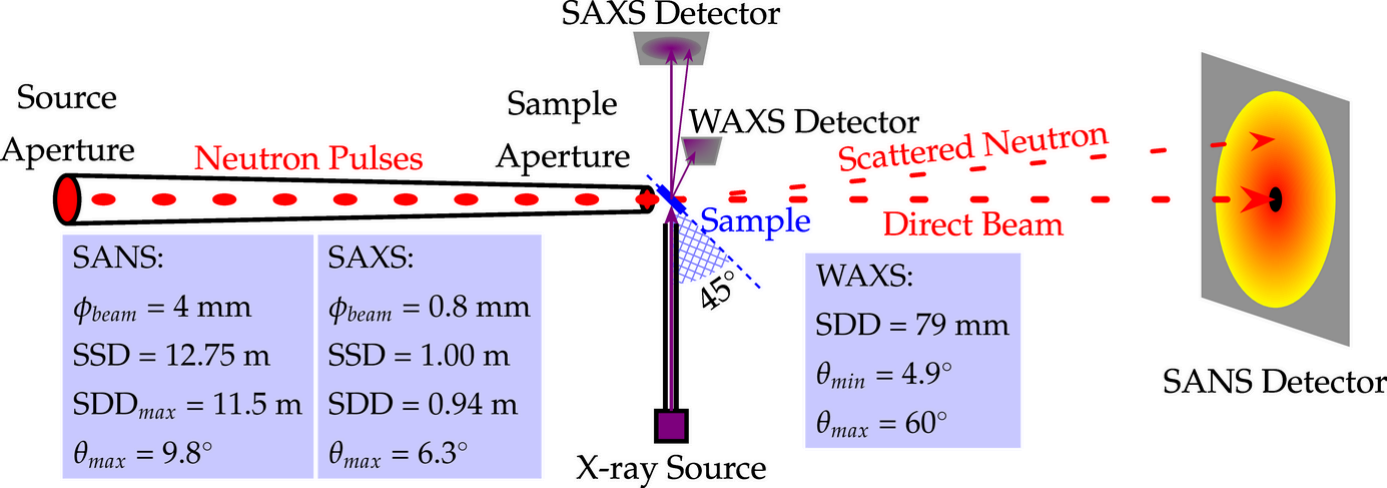
Schematic drawing of simultaneous SANS and SAXS.

In consideration of this, a pioneering vertical-geometry SAXS environment has been implemented at beamline 14, Very Small Angle Neutron Scattering (VSANS) instrument, at the China Spallation Neutron Source (CSNS) (Zuo *et al.*, 2024 ▸; Luo *et al.*, 2021 ▸; Zuo *et al.*, 2016 ▸). Following the removal of the sample stage within the sample chamber, a square fixing base is affixed with four dowel pins at each corner. The modified vertical-geometry SAXS instrument, accompanied by a hoisting frame, is then elevated onto the fixing base. Utilizing the four dowel pins, the SAXS instrument is securely mounted on the fixing base. A 50 mm-diameter collimation guide is inserted from the rear panel of the SAXS sample chamber to its designated sample position. Subsequently, a 48 mm-diameter sapphire window is inserted to seal the vacuum of the neutron collimation system. Next to it is a 4 mm sample aperture, which defines the sample cross section for the *in situ* SAXS measurement on the 45°-tilted sample holder. It also facilitates the convergence of the neutron and X-ray beams at the sample position. The complete installation process of the SAXS environment can be finalized within a timeframe of 5 h, followed by a series of comprehensive tests. A schematic view of the *in situ* SAXS measurement at the VSANS instrument is shown in Fig. 1 ▸.

## 2. The overall configuration of SAXS at VSANS

### 2.1. SAXS instrument

The primary instrumental factors of the SAXS instrument, such as size, neutron flux at the sample position and scattering vector magnitude (*Q*), need to align with the requirements of SANS. To achieve this, the SAXS setup needs to adopt a vertical geometry to minimize space utilization in the VSANS sample chamber. Additionally, as the SANS instrument employs pinhole collimation for obtaining 2D scattering patterns, the Kratky collimation geometry cannot be used for SAXS. The neutron flux at the sample position is 2.6 × 10^7^ n (s cm^2^)^−1^; therefore the SAXS flux should be comparable to allow a similar measurement time for typical samples. Furthermore, the range of the scattering vector magnitude (*Q*) for SAXS/WAXS must be similar to the *Q* range for SANS, which is between 0.0017 and 1.8 Å^−1^. In light of these requirements, the Xenocs Nano-inXider was selected as the SAXS instrument, as it has no beam stop and its key parameters are compatible with the SANS model at VSANS. The SANS and SAXS instrument parameters are compared in Table 1 ▸ (Zuo *et al.*, 2024 ▸; Zuo *et al.*, 2016 ▸).

### 2.2. Modifications implemented on the SAXS instrument

#### 2.2.1. Lifting and positioning of SAXS sample environment

**Figure 2:**
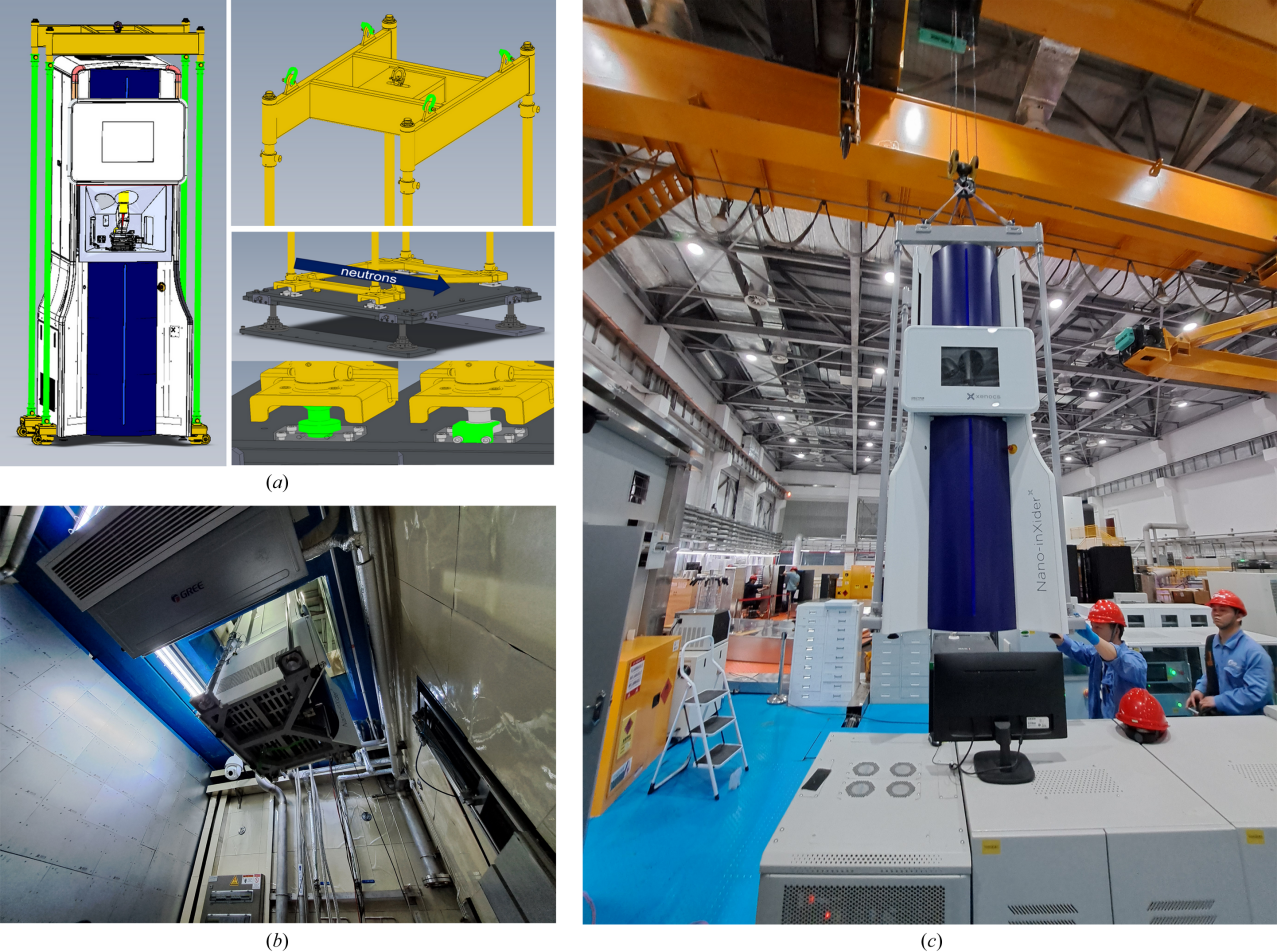
SAXS hoisting frame and hoisting process. (*a*) The design of the lifting frame. (*b*) and (*c*) The process of lifting the SAXS instrument from the top to the VSANS sample chamber.

The SAXS instrument has dimensions of roughly 87 × 93 × 240 cm^3^ (W × L × H) and weighs around 520 kg. To facilitate quick installation and positioning, a custom lifting frame measuring about 110 × 120 × 270 cm^3^ (W × L × H) was created. The frame comprises a base, four load-bearing columns and a top structure with a crane hook attachment. It is linked to the instrument using stainless steel pins which can be detached for standalone use. A 1.3 m × 1.3 m hoisting hole is situated at the top of the sample chamber for easy lifting, with a screen door installed to prevent neutron and γ photon leakage during experiments. Fig. 2 ▸ illustrates the hoisting frame and hoisting process.

To facilitate the rapid and precise placement of the X-ray sample environment within the neutron beam, the neutrons and X-rays are oriented perpendicularly to one another at the centre of the experimental specimen. A stainless steel platform has been installed on the floor of the sample chamber, enabling the SAXS instrument to be adjusted in three-dimensional space. Dowel pins with a diameter of 50 mm connect the X-ray sample environment to the platform, ensuring accurate positioning. During the initial setup, a laser tracker is employed to ascertain the position of the sample’s centre, confirming that both the neutron and the X-ray beams intersect at this central point with a positional deviation of less than 1 mm. Once alignment is finished, the platform screws are tightened to stabilize the XYZ positions, thereby maintaining the relative orientation between the X-ray and the neutron beams. For subsequent installations, only the alignment of the base and hoisting frame dowel pins is necessary to guarantee the precise positioning of the X-ray sample environment, thus expediting the installation process.

#### 2.2.2. The relative position between the X-ray and the neutron beams

**Figure 3:**
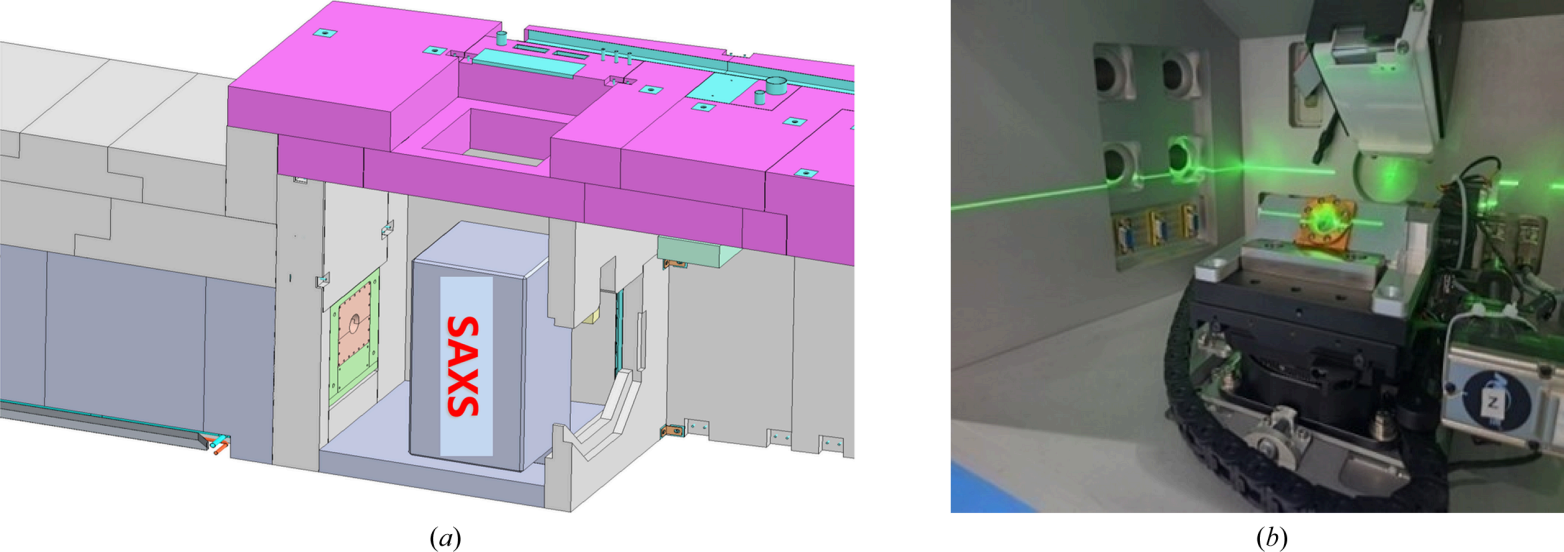
The placement of the SAXS instrument in the VSANS sample room (*a*). Employing a laser collimator to optimize the alignment of the SAXS sample’s central position (*b*).

The SAXS instrument is integrated into the sample room of the VSANS instrument [Fig. 3 ▸(*a*)]. X-rays are directed perpendicularly onto the sample from below, while small-angle and wide-angle X-ray detectors are positioned above to capture the scattered X-rays. Concurrently, the neutron beam is incident horizontally on the sample, with scattered neutrons being detected by a ^3^He tube neutron detector array located in the neutron detector vessel (Fig. 1 ▸). The X-ray and neutron beams are oriented orthogonally, and the sample is inclined at a 45° angle relative to the vertical axis, facilitating the simultaneous traversal of both X-rays and neutrons through the same sample region [Fig. 3 ▸(*b*)]. For SANS, there are three ^3^He detectors in the detector vessel, and their sample–detector distance (SDDs) are 1, 4 and 11.5 m, respectively; for SAXS, there is only one Pilatus detector, and its SDD is 0.94 m. Therefore, because of the 45° tilting, different sample-thickness corrections might be needed for SANS and SAXS in the data reduction process. However, because of the small sample area, this tilted-sample-induced SDD correction can be neglected.

**Figure 4:**
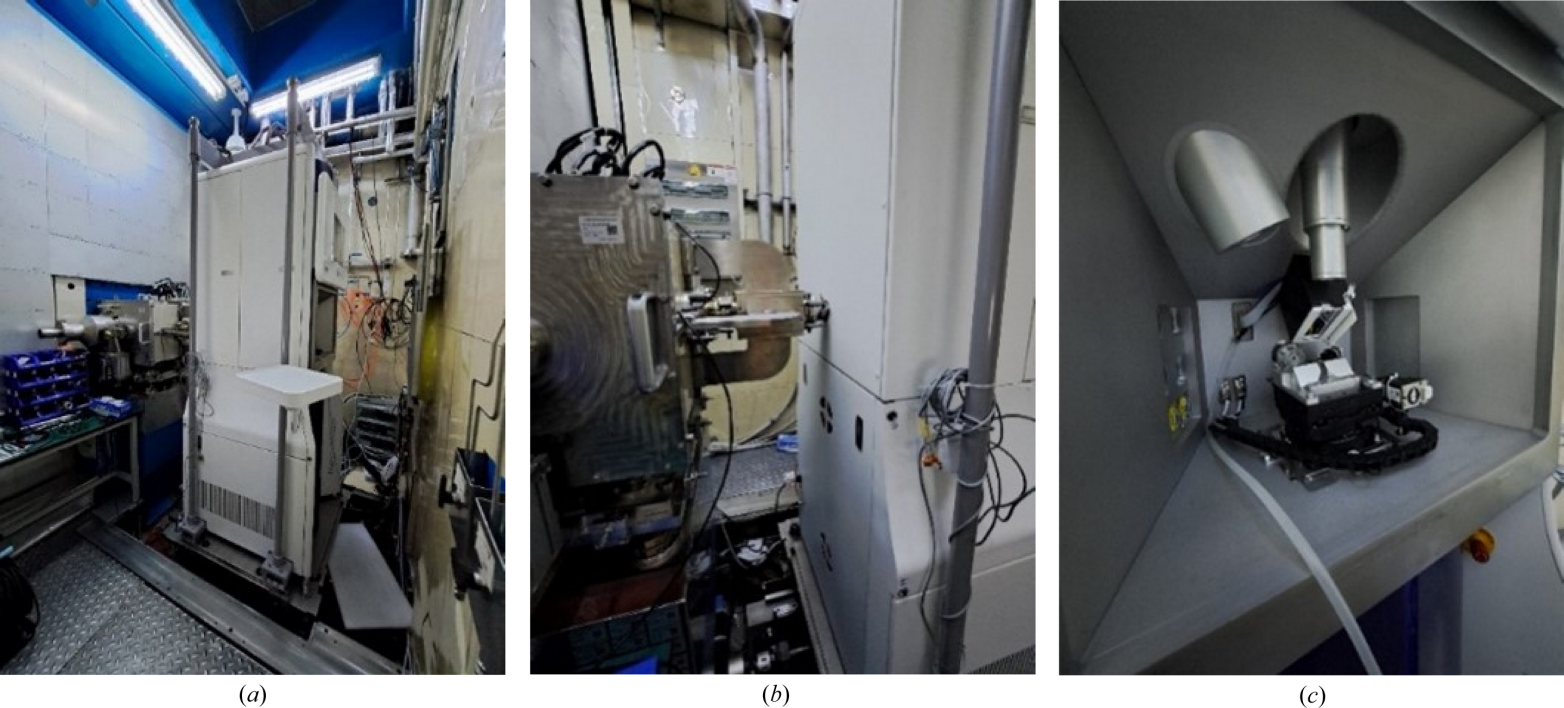
SAXS instrument in the sample room (*a*), neutron guide (*b*) and compressed air exhaust conduit for the wide-angle detector (*c*).

To minimize the neutron beam’s exposure to air and thereby reduce background scattering, we use a neutron guide with an outer diameter of 50 mm and an inner diameter of 5 mm, channelling the neutron beam toward the experimental sample as closely as possible [Fig. 4 ▸(*a*)]. A horizontal circular hole with a diameter of 60 mm is incorporated into the rear of the X-ray sample chamber to facilitate the passage of the neutron guide [Fig. 4 ▸(*b*)]. The SANS sample aperture is 4 mm, and it is 10 cm to the tilted sample position [Fig. 4 ▸(*c*)]. The 4 mm SANS sample aperture is the final aperture that determines the optics of the neutron scattering setup and the resolution.

#### 2.2.3. Sample holder and sample cell

**Figure 5:**
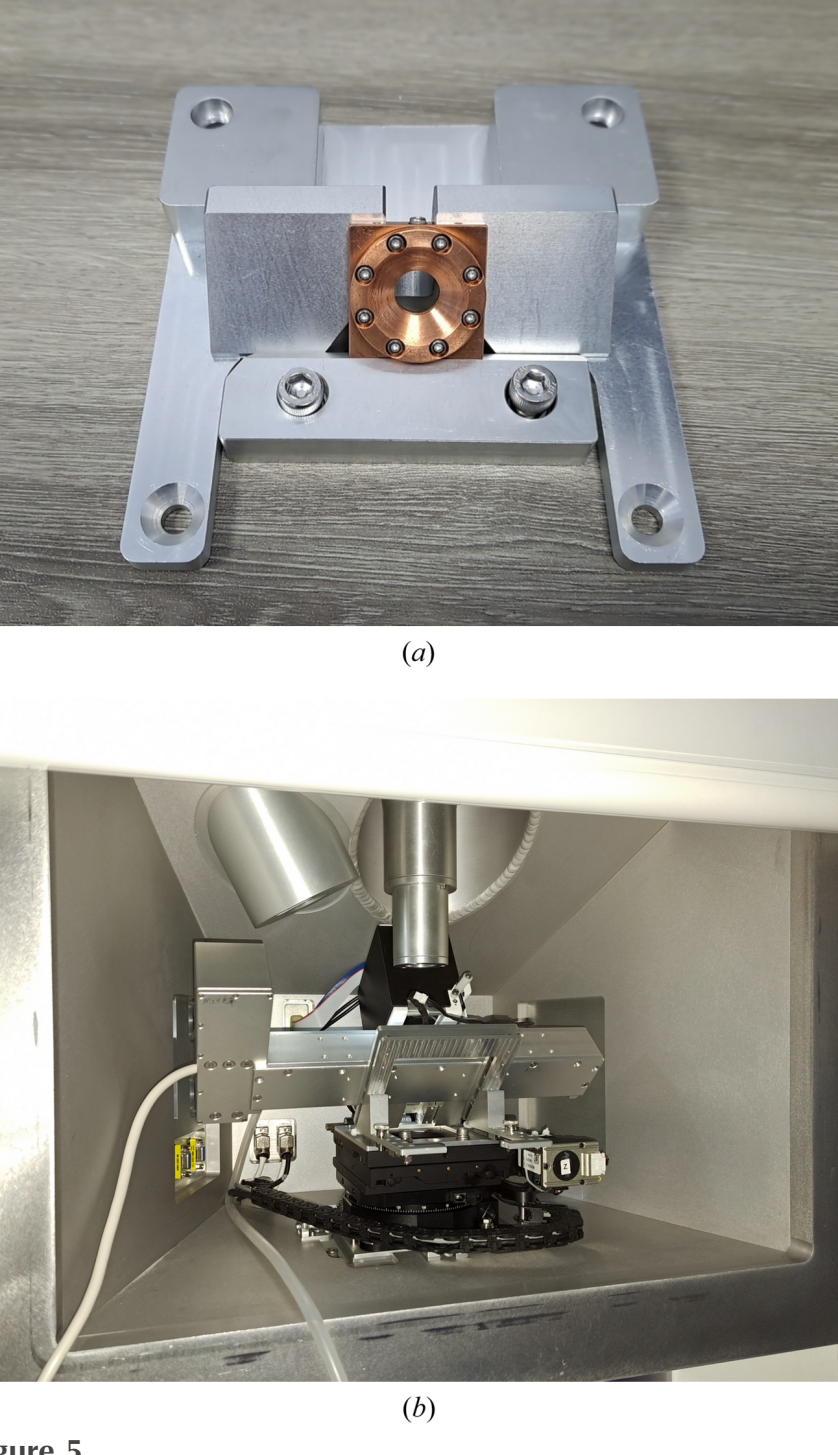
Sample stage and solution sample cell (*a*). Stretching machine with an inclination of 45° (*b*).

To achieve a 45° tilt of the sample in the vertical plane, we engineered the tilted sample holder and the SAXS sample chamber to accommodate the requirements of both neutron and X-ray scattering experiments simultaneously, as depicted in Fig. 5 ▸. For an X-ray experiment, the X-ray beam diameter at the sample position is kept below 1 mm, whereas for the simultaneous neutron experiment, the neutron beam diameter is set by the 4 mm sample aperture. Considering the 45° inclination of the sample and a potential deviation of the neutron beam centre of approximately 1 mm, we designed the effective cross-sectional area at the tilted sample holder to have a diameter of 10 mm. This configuration ensures that both neutrons and X-rays can traverse the experimental sample entirely without obstruction from the SAXS chamber’s support structure, and neutrons with scattering angles below 9.8° are unobstructed by the supporting components. For liquid samples, we have developed specialized cells designed to hold the solution, featuring 25 µm mica sheets affixed on either side by a copper frame and rubber seals. The resulting sample chamber has a thickness of 1 mm and a diameter of 1.5 cm, along with a 0.7 mm-diameter filling port for syringe-based sample injection (Note: copper has an activation problem, and we will replace copper with titanium in the future.) Fig. 5 ▸(*a*) shows the sample holder and solution cell. For solid samples, we position the sample directly against the tilted holder to maintain the required angle of inclination. To measure an elongated sample, we also designed a sample-stretching environment. Simultaneous SAXS and SANS microstructure evolution at different stretch ratios can be recorded [Fig. 5 ▸(*b*)].

#### 2.2.4. Safety interlocks

To allow the synchronization of measurements with neutron scattering, the protective access door to the X-ray scattering sample chamber remains open. To maintain radiation safety for the experimenter, the shutter of the SAXS instrument is securely interlocked with the safety door of the VSANS sample chamber. This safety gate provides two dry contact signals (X1 and X2) that convey the opening and closing status of the safety gate to the SAXS instrument. The SAXS instrument is permitted to activate the light source shutter and commence experimental data acquisition only when both dry contact signals are simultaneously enabled. Conversely, if either of the dry contact signals is interrupted, the instrument will be unable to activate the light shutter. In the event that an X-ray scattering experiment is underway, should the safety door be unexpectedly opened, the SAXS instrument will promptly deactivate the light source and halt the experiment. Additionally, the SAXS instrument transmits two parallel dry contact signals (Y1, Y2) to indicate the status of the X-ray light source shutter to the safety door, ensuring that the neutron sample chamber’s safety door cannot be opened while either dry contact signal remains active, thereby safeguarding users from X-ray radiation exposure. A schematic diagram of the safety interlocking is shown in Fig. S1.

## 3. Instrument calibration and validation

### 3.1. Sample position calibration

In the SAXS chamber, the inclined stage utilized in conjunction with neutrons exhibits a slight height variation from the conventional stage. Thus it is imperative to calibrate the position of the SAXS detector with respect to the sample’s centre using standard samples. The positions of the wide-angle and small-angle SAXS detectors are calibrated in relation to the centre of the sample using LaB_6_ and silver behenate, respectively. Post-calibration assessments reveal an elevation of approximately 3 mm in the sample height compared with the standard sample holder, along with an increase of about 2° in the tilt angle of the wide-angle detector. The test results of the characteristic peaks of the LaB_6_ and silver behenate samples after calibration coincide with the theoretical values (Figs. S2 and S3). The calibrated positional parameters of the detectors are subsequently input into the X-ray sample environment prior to conducting the SANS experiment.

### 3.2. Standards test

We evaluated the performance of the instrument by utilizing glassy carbon (SRM3600) and deuterated and hydrogenated polyethylene glycol (PEG-d4 and PEG-h4). The PEG-d4 is full deuterated polyethylene with Mn 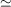 2400 g mol^−1^ and Mw/Mn = 1.08. The PEG-h4 is fully hydrogenated polyethylene with Mn 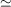 4000 g mol^−1^ and Mw/Mn = 1.10 The samples were prepared by hot-pressing at 70°C and measured at room temperature. We used the direct-beam method to reduce experimental data to obtain the absolute intensity of neutron scattering (Zuo *et al.*, 2024 ▸). Using a small sample aperture of 1 mm, we first measured the direct beam of the sample, the empty cell and the vacuum separately to obtain their direct-beam intensities 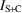, 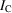 and 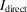, respectively. Then we tested the scattering of the sample and the empty sample cell with a normal sample aperture of 4 mm, to obtain the scattered neutron counts at the neutron detector. The absolute intensity of neutron scattering of the sample is calculated by the following formula: 
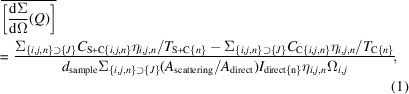
where 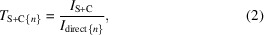

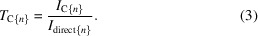
Σ is the macroscopic scattering cross section, Ω is the solid angle, *C* denotes neutron counts, *d*_sample_ is the thickness of the sample, and 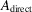 and 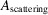 are the areas of the sample aperture used in the direct beam and in the scattering measurements. {*i*, *j*, *n*} 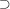 {*J*} means accumulating the neutron counts at all detector pixels (*i* and *j* label the detector pixel in the 2D neutron detector) and with all neutron wavelengths (*n* labels the different wavelengths). 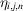 is the efficiency of the detector pixel (*i*, *j*) at neutron wavelength 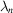.

**Figure 6:**
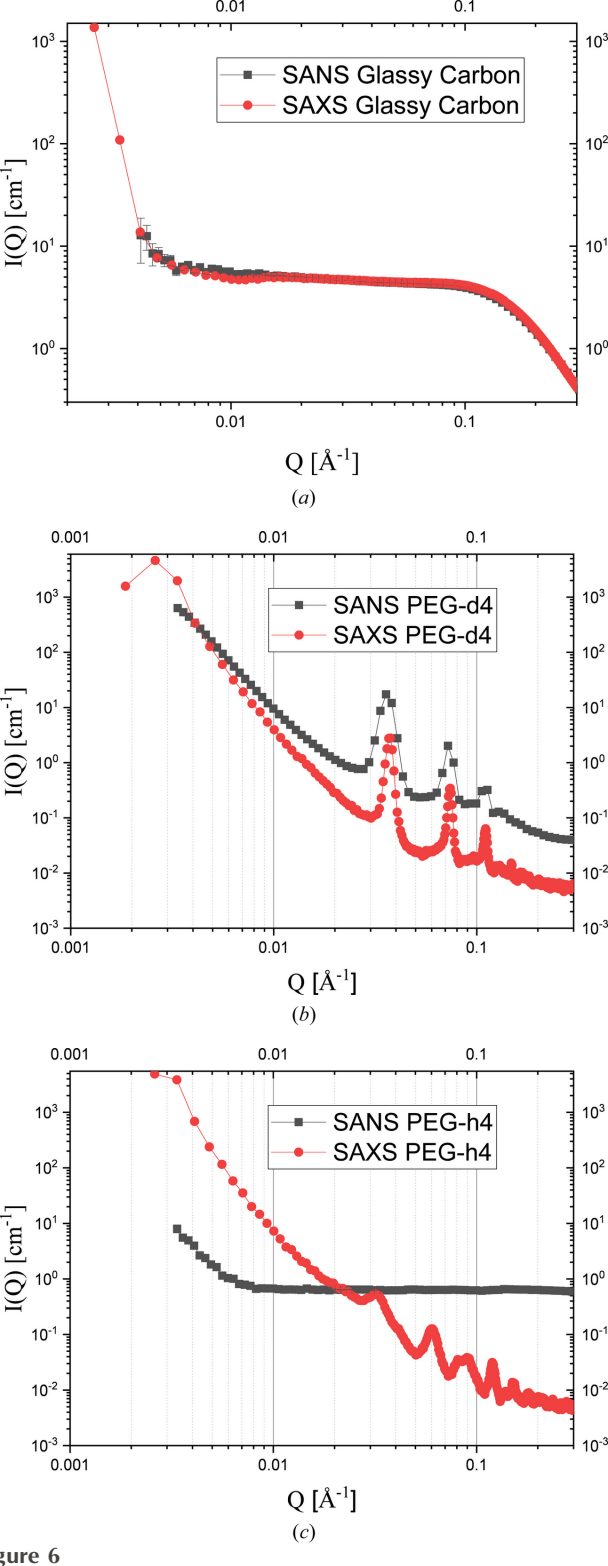
SAXS and SANS profiles of glassy carbon (*a*), PEG-d4 (*b*) and PEG-h4 (*c*) in the simultaneous SAXS and SANS tests.

Fig. 6 ▸ depicts the one-dimensional scattering profiles of the standard samples. After the SAXS data have been scaled, the SAXS and SANS scattering patterns of glassy carbon coincide [Fig. 6 ▸(*a*)] (Zuo *et al.*, 2024 ▸; Allen *et al.*, 2017 ▸). For the crystalline polymer, both the scattered neutron and X-ray intensities are proportional to the square of the difference of the scattering length density between the crystalline and amorphous lamellae. In SANS, the scattering length of CH_2_ is almost zero, while that of CD_2_ is 19.8 fermi. Therefore, the SANS scattering profile of PEG-d4 is similar to its SAXS scattering profile, and the characteristic peaks coincide, but the SANS scattering profile of PEG-h4 is a flat background. Fig. 6 ▸ also demonstrates the importance of the simultaneous SANS and SAXS measurements in the polymer field. We can take the temperature dependence of multi-scale structure evolution in a polymer alloy during stretching as an example. SAXS can monitor crystallization, SANS can see the phase separation, and the combination of these two can reveal what happens first when we assume that SAXS and SANS have similar fluxes at the sample position and their detectors have similar efficiencies.

## 4. Conclusion

Small-angle X-ray and neutron scattering (SAXS and SANS) are pivotal techniques for probing the nanoscale architecture of materials. Typically, SAXS exploits the electron density contrast between nanoparticles and their surrounding solvents to elucidate the overall morphology of nanoparticles, whereas SANS can study the structure of different parts of nanoparticles by contrast matching. Consequently, SAXS and SANS exhibit significant complementary strengths. A simultaneous online measurement of both SAXS and SANS will ensure that the samples in the two scattering experiments are identical, facilitating coherent data analysis and enabling the extraction of more comprehensive structural information regarding the experimental specimens. Inspired by the ILL D22 spectrometer, we have integrated a SAXS apparatus into the very small angle spectrometer at CSNS. We have successfully conducted simultaneous measurements of X-ray scattering and neutron scattering. Utilizing deuterated and hydrogenated PEG as test samples, we have obtained their unique scattering signals.

Because of the weak X-ray penetration ability, the combined SAXS and SANS measurement have two main limitations. The first limitation is air scattering. To avoid it, we can only measure a strong scattering sample or keep the sample under vacuum. The second limitation is the weak X-ray beam flux. Compared with synchrotron radiation, the flux of the Nano-inXider is weak. It thus cannot see the protein conformation in dilute solution.

The current scattered neutron signal from the VSANS D1 neutron detector is compromised by a spatial discrepancy (SDD) of approximately 1 m between the sample location in the X-ray scattering apparatus and the designated position for neutron scattering. During concurrent X-ray measurements, the neutron scattering *Q* range is limited, reaching a maximum of merely 0.3 Å^−1^. We plan to overhaul the base of the X-ray instrument by integrating stepper motors that facilitate forward and backward movement. This modification will not only streamline the sample replacement process but also allow the experimental sample to be positioned as close to the neutron detector as feasible, thereby preventing obstruction of neutrons directed towards the D1 detector and enabling a broader *Q* measurement range.

## Supplementary Material

Supporting information file. DOI: 10.1107/S1600576725001232/jl5097sup1.pdf

## Figures and Tables

**Table 1:** Comparison of SANS and SAXS parameters

Parameter	SANS		SAXS	
Wavelength (Å)	2.2–6.7		1.54	
Scattering vector range (Å^−1^)	0.0016–1.8		0.0029–4.1	

Flux in different collimation modes:	2.49 m	26	VHR (very high resolution)	4
SANS: 10^6^ n (cm^2^ s)^−1^ at 100 kW	5.15 m	9.0	HR (high resolution)	15
SAXS: 10^6^ photons s^−1^	9.92 m	2.0	MR (medium resolution)	60
	12.75 m	1.6	VHF (very high flux)	100

Detectors	^3^He tube array detectors		Dectris Pilatus 3	
	Diameter 8 mm		Detector area 83.8 × 33.5 mm^2^	
			Pixel size 172 µm	
Sample size (mm)	∼4, 6, 8		<1	
