# Supplementary material for: In situ small-angle X-ray scattering measurement at the Very Small Angle Neutron Scattering Instrument at the China Spallation Neutron Source
Source: J Appl Crystallogr. 2025 Feb 28;58(Pt 2):573–80. doi: 10.1107/S1600576725001232 (PMC11957413; doi:10.1107/S1600576725001232)
Supplement: Supplementary file 1 [file j-58-00573-sup1.pdf]

Supporting Information

Safety interlock control circuit diagram

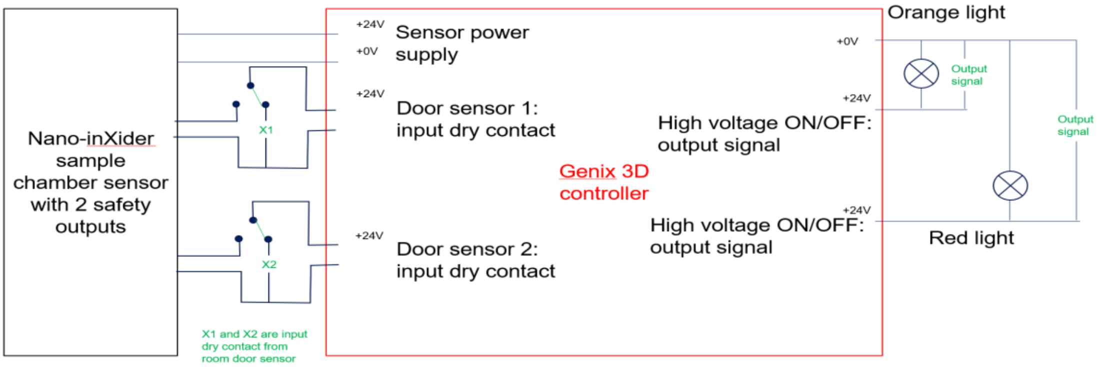

Figure S1. Safety interlock control circuit diagram

The detector position calibration of the wide-angle and small-angle X-ray scattering with LaB6 and Silver behenate.

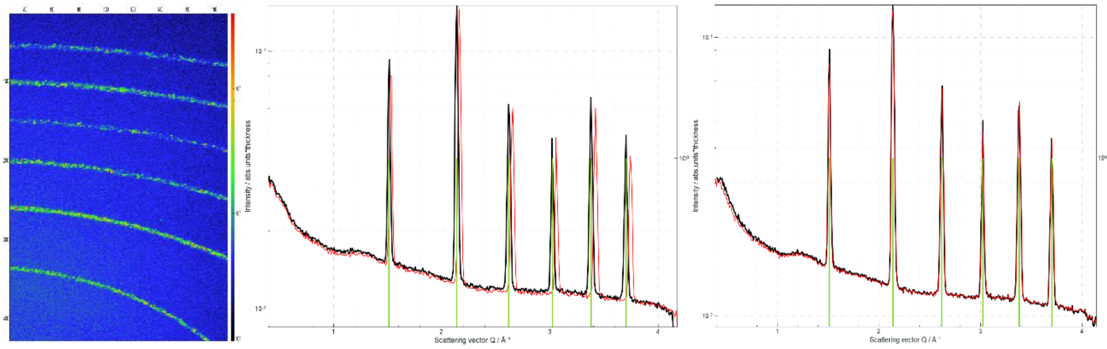

Figure S2. 2D WAXS profile of LaB6 (left), LaB6 sample's peak shift before detector position calibration (middle) and after detector position calibration (right).

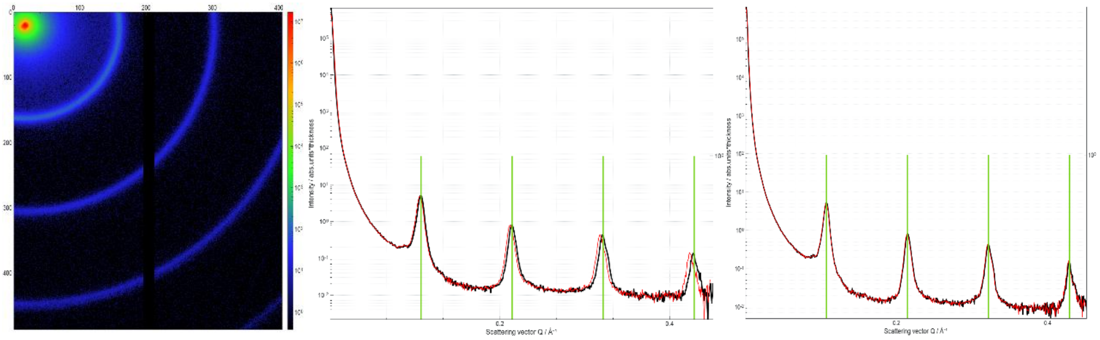

Figure S3. 2D SAXS pattern of silver behenate (left), silver behenate's peak shift before detector position calibration (middle) and after detector position calibration (right).
